# Meetings of Societies

**Published:** 1951-01

**Authors:** 


### Meetings of Societies

### Bristol Medico-Chirurgical Society

The Annual General Meeting was held in the University building on Wednesday, October 11th. Mr. W. A. Jackman gave his Presidential Address (p. 1).

### *Officers for* 1950

*President:* Mr. W. A. Jackman. *President-Elect:* Dr. Victoria Tryon.* *Treasurer:* Dr. G. E. F. Sutton. *Hon. Secretary:* Mr. A. L. Eyre Brook. *University Library:* Professors C. Bruce Perry, Milnes Walker and H. J. Drew Smythe. *Editor:* Mr. E. Watson-Williams. *Assistant Editor:* Dr. N. S. Craig.

*Committee:* Drs. Bain,* Beryl Corner,* MacLachlan,* Marwood, J. E. Lucas, Silvey, Gornal, Kersley.

The Honorary Secretary read his report for the past year, which was adopted with thanks: the membership of the Society reached 395. The Honorary Treasurer's report is embodied in the balance sheets on pp. 28, 29.

### *Editor's Report*

Last year it was hoped that we might soon be able to relax at least some of the restrictions imposed by the need for economy. This hope faded before it appeared in print. Instead, during 1950, we have had considerable financial anxieties, owing to the great increase in charges for printing and publishing which came into effect in January. Unfortunately, most of our advertisement contracts had already been made for the year, and profits from advertisements were therefore small. By severe pruning and other economies we have just managed to pay our way: and desire again to express thanks to the contributors who so cheerfully submitted to the necessary, but probably none the less irksome, restrictions.

We have tried to do justice to the work of many local societies, but it seems doubtful whether we can continue this policy, unless we get more non-member subscriptions to the *Journal*. As already announced, the number of readers interested in " Reviews of Books " no longer justifies the retention of this feature. During the year we received for review thirty-nine books, of which the published price was £50; these have been handed over to the Medical Library, together with eighty-nine medical periodicals, priced at £114, received in exchange for the *Journal*. This sum of £164 is a contribution to the Library from readers of the *Journal*.

*Elected 1950.

### BRISTOL MEDICAL-CHIRURGICAL SOCIETY

### INCOME AND EXPENDITURE ACCOUNT for the year ended September 30th, 19[illegible]

### EXPENDITURE

| | | £ | s. | d. |
|---|---|---|---|---|
| To Subscriptions to *Medico-Chirurgical Journal* ... ... ... ... ... | | 307 | 4 | 0 |
| ,, Grant to Medical Library | £25 0 0 | | | |
| ,, Lewis's Medical and Scientific Library ... | 8 9 11 | | | |
| | | 33 | 9 | 11 |
| ,, Librarian, Honorarium ... ... ... | | 15 | 0 | 0 |
| ,, Treasurer's Secretary Honorarium ... | | 10 | 0 | 0 |
| ,, Lecturer and Transport of Patients ... | | 12 | 13 | 0 |
| ,, Audit Fee ... ... ... ... ... | | 8 | 8 | 0 |
| ,, Printing and Stationery ... | £39 16 11 | | | |
| ,, Printing Laws of the Society | 19 15 0 | | | |
| | | 59 | 11 | 11 |
| ,, Catering ... ... ... ... ... | | 78 | 17 | 6 |
| ,, Deficit on Reunion Dinner ... ... | | 43 | 11 | 1 |
| ,, Other Expenses *re* Meetings ... ... | | 7 | 7 | 0 |
| ,, Postages and Sundries ... ... ... | | 29 | 16 | 9 |
| ,, Surplus for the Year ... ... ... | | 23 | 16 | 2 |
| | | £629 | 15 | 4 |

### INCOME

| | £ | s. | d. |
|---|---|---|---|
| By Members' Subscriptions ... ... ... | 605 | [illegible] | [illegible] |
| ,, Interest on Bank Deposit Account ... | 1 | [illegible] | [illegible] |
| ,, Refund by *Journal* ... ... ... (See *Journal* LXVII, 242, p. 62 footnote) | 22 | 17 | [illegible] |
| | £629 | [illegible] | [illegible] |

### BALANCE SHEET, September 30th, 1950

### LIABILITIES

| | | | £ | s. | d. |
|---|---|---|---|---|---|
| Sundry Creditors ... ... ... ... | | | 53 | 13 | 3 |
| Subscriptions paid in advance ... ... | | | 1 | 11 | 6 |
| Cash due to the *Journal* ... ... ... | | | 359 | 8 | 2 |
| Accumulated Fund, Sept., 1949 | | £532 16 2 | | | |
| *Add:* | | | | | |
| Surplus on realization of £500 4 per cent. Consuls ... | | 85 9 6 | | | |
| Surplus for year 1949–50 ... | | 23 16 2 | | | |
| | | 642 1 10 | | | |
| *Deduct:* | | | | | |
| Arrears of Subscriptions written off | £113 6 6 | | | | |
| Payment to *Journal* (*q.v.*) ... | 270 0 0 | | | | |
| | | 383 6 6 | | | |
| | | | 258 | 15 | 4 |
| | | | £673 | 8 | 3 |

### ASSETS

| | £ s. d. | £ | s. | d. |
|---|---|---|---|---|
| Cash at Bank: | | | | |
| Current Account ... ... | 62 0 7 | | | |
| Deposit Account ... ... | 443 15 8 | | | |
| | | 505 | [illegible] | [illegible] |
| Arrears of Subscriptions due ... ... | | 167 | [illegible] | [illegible] |
| | | £673 | [illegible] | [illegible] |

13 Meadow Street, Weston-super-Mare,
*October 6th,* 1950.

Audited and found correct.
C. J. RYLAND & CO., *Chartered Account[illegible]*

### BRISTOL MEDICO-CHIRURGICAL JOURNAL

### INCOME AND EXPENDITURE ACCOUNT for the year ended September 30th, 1950

### EXPENDITURE

| | | £ s. d. | £ s. d. |
|---|---|---|---|
| Arrowsmith, Printing: | | | |
| January, 1950 ... ... | | 160 19 2 | |
| April ... ... ... ... | | 128 2 8 | |
| July ... ... ... ... | | 149 15 10 | |
| October (estimate) ... ... | | 150 0 0 | |
| | | 588 17 8 | |
| October, 1949 (estimate) ... | £152 11 4 | | |
| Cost ... ... | 127 7 10 | | |
| Deduct ... ... ... | | 25 3 6 | |
| | | | 563 14 2 |
| Auditors ... ... ... ... ... | | | 3 3 0 |
| | | | 566 17 2 |
| Surplus for 1950 ... ... ... ... | | | 257 16 8 |
| | | | £824 13 10 |

### INCOME

| | | | £ s. d. |
|---|---|---|---|
| Subscriptions: | | | |
| Members of Bristol Med. Chir. Society* ... | | | 307 4 0 |
| ,, Torquay Med. Chir. Society | | | 15 10 0 |
| Non-members ... ... ... ... | | | 12 12 6 |
| Subscriptions due 1940–45 ... | | £100 4 1 | |
| Grant for 1946 ... ... | | 169 15 11 | |
| Total Voted in 1947 ... ... | | | 270 0 0 |
| Advertisements, 1950: | | | |
| January ... ... ... | | 53 1 6 | |
| April ... ... ... ... | | 58 18 9 | |
| July ... ... ... ... | | 55 13 2 | |
| October (estimate) ... ... | | 50 0 0 | |
| 1949, October: | | | |
| Received ... | £50 13 5 | | |
| Estimated ... | 48 19 6 | | |
| *Add* ... ... ... | | 1 13 11 | |
| | | | 219 7 4 |
| | | | £824 13 10 |

### BALANCE SHEET, September 30th, 1950

### LIABILITIES

| | | £ s. d. | £ s. d |
|---|---|---|---|
| Arrowsmith for Printing: | | | |
| Due September, 1949 ... | | 372 10 6 | |
| For 1950 ... ... ... | | 563 14 2 | |
| | | 936 4 8 | |
| Advertisements | £219 7 4 | | |
| Cash Paid ... | 222 10 6 | | |
| Deduct ... | | 441 17 10 | |
| | | | 494 6 10 |
| Auditors ... ... ... ... ... | | | 3 3 0 |
| | | | 497 9 10 |
| Surplus for 1950 ... ... | | 257 16 8 | |
| Deficit September, 1949 ... | | 95 2 5 | |
| Surplus September, 1950 ... | | | 162 14 3 |
| | | | £660 4 1 |

### ASSETS

| | £ s. d. |
|---|---|
| Cash at Bank ... ... ... ... ... | 290 15 11 |
| ,, due from Bristol Med. Chir. Society ... | 359 8 2 |
| ,, ,, ,, Torquay Med. Chir. Society | 10 0 0 |
| (These sums included in " Income " above) | |
| | £660 4 1 |

*Note.* In addition we have received, too late for inclusion in the Balance Sheet, the sum of £26 15s 6d. for miscellaneous sales during 1945-50.

Audited and found correct.
C. J. RYLAND & CO., *Chartered Accountants.*

* It is not clear why only 384 subscriptions have been received although the *Journal* was delivered to 395 members.

The subjoined analysis shows very clearly that the only factor of any importance in the cost of production is the number of words—or pages. This may explain our almost morbid desire for terseness.

| No. | Pages | Typesetting | | | Other Costs | | | Total Costs | | | Received for Advertisements | | |
|---|---|---|---|---|---|---|---|---|---|---|---|---|---|
| | | £ | s. | d. | £ | s. | d. | £ | s. | d. | £ | s. | d. |
| 241, January .. | 41, xv | 97 | 10 | 0 | 63 | 9 | 2 | 160 | 19 | 2 | 53 | 1 | 6 |
| 242, April .. | 24, xvii | 64 | 15 | 9 | 63 | 6 | 11 | 128 | 2 | 8 | 58 | 18 | 9 |
| 243, July .. | 37, xvi | 87 | 1 | 0 | 62 | 14 | 10 | 149 | 15 | 10 | 55 | 13 | 2 |
| 244, October .. | 25, 4, xv | 69 | 8 | 0 | 56 | 2 | 4 | 125 | 10 | 4 | 54 | 14 | 0 |
| LXVII, 1950 | 127, lxiii | £318 | 14 | 9 | £245 | 13 | 3 | £564 | 8 | 0 | £222 | 7 | 5 |

The Second Meeting of the session was a clinical meeting at the Bristol General Hospital on November 8th. The following cases were shown: pituitary necrosis, acromegaly, macrocytic anaemia, cystitis, Reiter's syndrome (urethritis, etc.), keratosis pharyngis, temporal arteritis, congenital heart disease, mycosis fungoides, psoriasis, rhadritis, neonatal meningitis, Sturge-Kalesche-Weber disease, haemorrhagic telangiectasis, angina pectoris (relieved by operation), intrathoracic goitre, gout; and many X-ray films.

April, 1951.—Mr. Howard K. Gray: "**Surgical Trends in America**".

May.—Dr. John Atley and Professor Milnes Walker: "**Diagnosis and Treatment of Congenital Heart Disease**".

### Devon and Exeter Medico-Chirurgical Society

On October 26th Mr. Alexander gave an address on "**The Scope of Neuro-Surgery**", which we hope to publish in our next issue.

### Plymouth Medical Society

*Calendar*, 1951

Meetings at 8.30 p.m.

January 12th. Short papers.

February 2nd and 25th. Clinical Meetings.

February 16th. Professor R. Milnes Walker, M.S., F.R.C.S., "**The Spleen**".

Wednesday, March 16th, at 3.30 p.m. Clinical demonstration at Didworthy Sanatorium.

Mr. R. C. Brock will address the Plymouth Medical Society during the winter. The full fixture list will be published later.

### British Medical Association

### Gloucestershire Branch

*Programme for* 1951

January 11th, at Cheltenham. Mr. J. S. Robinson, F.R.C.S.: "**Injuries and Derangements of the Knee Joint**".

February 8th, at Gloucester. Subject to be announced.

March 8th, at Cheltenham. Mr. Ronald Belsey, Esq., M.S., F.R.C.S.: "**The Surgical Causes of Dysphagia**".

April 12th, at Gloucester. Clinical Meeting.

May 10th, at Stroud. Annual Meeting. Dr. A. Fewster Blake: "**Sixty Years' Retrospect**".

June 14th, at Gloucester. The President's Guest.

*Officers for* 1950–51

*President:* J. F. H. Stallman, F.R.C.S. *President-Elect:* H. Sandeman Allen, F.R.C.S. *Vice-President:* Dr. J. Greene, *D.S.O.*, *M.C.* *Hon. Secretary:* Dr. E. W. Hyde (Royal Infirmary, Gloucester).

*Council:* R. G. Anderson, A. B. Cardew, C. Cookson, E. N. Davey, J. H. Grove-White, F. G. Lidderdale, F. C. Logan, J. C. Marklove, S. L. Marklove, R. W. H. Tincker, W. J. Wilkin, F. H. Woolley.

### Bath, Bristol and Somerset Branch

*President :* Dr. W. J. Petty

*Hon. Secretary :* Dr. K. C. Bailey

Programme, 1951

February 7th, 1951.—Meeting at Bath.

April 4th, 1951.—Clinical Meeting at Bristol.

May 3rd, 1951.—Meeting at Taunton.

### West of England and Wales Society of Dermatology

The Autumn meeting of the above Society was held in Bristol on Saturday, October 14th. Dr. R. P. Warin was in the Chair: seventeen members attended and the Society welcomed as its guest Dr. J. T. Ingram. Twenty-one cases were shown by Drs. C. D. Evans, R. P. Warin, E. Prettejohn, M. Hewitt, B. Brosnan and Sybil Jacob. These included: lipoid rheumatism (Parkes Weber); granulosis rubra nasi; Fox Fordyce disease in a male; recurrent herpetiform dermatitis repens; adenoma sebaceum (Pringle); erythema annulare centrifigum; dermatitis repens.

Future meetings, 1951: January 27th, at Newport; May 12th, at Plymouth.

South-Western Laryngological Association

On Saturday, October 14th, a very full meeting of this association was held at Exeter, in the pleasantly unspoiled surroundings of the Royal Devon and Exeter Hospital. Mr. Philip Scott and Mr. Hine provided a very well-balanced and interesting series of cases. In addition to a number of patients showing the successful result of operation for mastoid disease with intracranial complications, and for cancer of the larynx and of the sinuses, there were several cases of laryngral and other diseases for diagnosis, which provided material for a lively discussion. The next meeting will be in Bristol on January 6th.

